# Deciphering the Prognostic and Therapeutic Significance of Cell Cycle Regulator CENPF: A Potential Biomarker of Prognosis and Immune Microenvironment for Patients with Liposarcoma

**DOI:** 10.3390/ijms24087010

**Published:** 2023-04-10

**Authors:** Jiahao Chen, Yingying Lian, Binbin Zhao, Jiayang Han, Xinyu Li, Jialin Wu, Mengwen Hou, Man Yue, Kaifeng Zhang, Guangchao Liu, Mengjie Tu, Weimin Ruan, Shaoping Ji, Yang An

**Affiliations:** 1 Cell Signal Transduction Laboratory, Department of Biochemistry and Molecular Biology, School of Basic Medicine, Bioinformatics Center, Henan University, Kaifeng 475004, China; 2 Kaifeng Key Laboratory of Cell Signal Transduction, Henan Provincial Engineering Center for Tumor Molecular Medicine, Kaifeng 475004, China; 3 Henan Key Laboratory of Brain Targeted Bio-Nanomedicine, School of Life Sciences & School of Pharmacy, Henan University, Kaifeng 475004, China; 4 Henan-Macquarie University Joint Centre for Biomedical Innovation, School of Life Sciences, Henan University, Kaifeng 475004, China

**Keywords:** liposarcoma, CENPF, cell cycle, prognostic biomarker, immune infiltration

## Abstract

Liposarcoma (LPS) is one of the most common subtypes of sarcoma with a high recurrence rate. CENPF is a regulator of cell cycle, differential expression of which has been shown to be related with various cancers. However, the prognostic value of *CENPF* in LPS has not been deciphered yet. Using data from TCGA and GEO datasets, the expression difference of CENPF and its effects on the prognosis or immune infiltration of LPS patients were analyzed. As results show, *CENPF* was significantly upregulated in LPS compared to normal tissues. Survival curves illustrated that high *CENPF* expression was significantly associated with adverse prognosis. Univariate and multivariate analysis suggested that *CENPF* expression could be an independent risk factor for LPS. *CENPF* was closely related to chromosome segregation, microtubule binding and cell cycle. Immune infiltration analysis elucidated a negative correlation between *CENPF* expression and immune score. In conclusion, *CENPF* not only could be considered as a potential prognostic biomarker but also a potential malignant indicator of immune infiltration-related survival for LPS. The elevated expression of *CENPF* reveals an unfavorable prognostic outcome and worse immune score. Thus, therapeutically targeting CENPF combined with immunotherapy might be an attractive strategy for the treatment of LPS.

## 1. Introduction

Liposarcoma (LPS), derived from adipocytes, is a slow-growing, painless and non-ulcerative swelling disease [1]. As one of the most common subtypes of sarcoma, LPS has an average annual incidence of 0.5 to 0.9 per 100,000 population [2]. The classification of LPS includes five histologic subtypes: well-differentiated/atypical lipomatous tumor (WDLPS); dedifferentiated liposarcoma (DDLPS); myxoid (round cell) liposarcoma (MRCLPS); pleomorphic liposarcoma (PLPS); and myxoid liposarcoma (MLPS) [3]. Surgical excision is the therapeutic strategy for most LPS, and radiation therapy is applied for treating larger primary extremity limb tumors as preoperative adjuvant therapy [4,5]. Although these treatments provide good controls of the local lesions, remote recurrence occurs in 40% to 50% of patients indicating that the disease is progressing [6]. In addition to traditional treatment methods, targeted therapy is gradually applied in clinical practice [7]. However, bridging the gap in terms of therapeutic needs, even though several potential biomarkers have been proposed to monitor LPS progression, such as *miR-145*, *miR-451* [8], *MDM2* [9] and *CDK4* [10], is still required. Therefore, it is of great significance to explore more robust candidate risk predictors for LPS to assist molecular targeted therapy for LPS patients.

Centromere protein F (CENPF) is a mammalian mitogenic protein located on human chromosome 1q41 [11]. CENPF, as a checkpoint protein, is essential for establishing kinetochore–microtubule attachment, thus involved in the regulation of mitosis and cell cycle progression [12]. The expression of CENPF is gradually accumulating until reaching its highest level in G2 and M phases and degrades rapidly after mitosis is complete [13]. Accordingly, CENPF regulates the cell cycle by activating CDK1 contributing to the completed mitosis and accelerated cell proliferation [14]. Recently, high expression of *CENPF* has been shown to be associated with the prognosis of various cancers. In particular, *CENPF* overexpression is significantly associated with tumor metastasis and poor prognosis of hepatocellular carcinoma or breast cancer patients [15,16]. In addition, *CENPF* was also found to be remarkably upregulated in pancreatic cancer, non-small cell lung cancer and papillary thyroid cancer and is commonly associated with shorter survival time [17,18,19]. However, up to now, as a major modulator of the cell cycle, the prognostic and therapeutic value of CENPF in LPS remains unclear.

In the present study, we aimed to elucidate the expression features of *CENPF* in LPS and the effects of *CENPF* expression on the prognosis of LPS patients, and then to speculate the roles of *CENPF* in the development of LPS and relevant mechanisms, and finally to explore the potential relationship between *CENPF* and the tumor immune microenvironment. *CENPF* might be a promising prognostic biomarker acting effectively in the diagnosis and treatment of LPS. Our findings will provide a better understanding of the mechanism underlying LPS tumorigenesis and progression, which will potentially facilitate the development of effective molecular targeted therapy strategies for LPS.

## 2. Results

### 2.1. Cell Cycle-Related Gene CENPF Is upregulated in Liposarcoma

**Figure 1:**
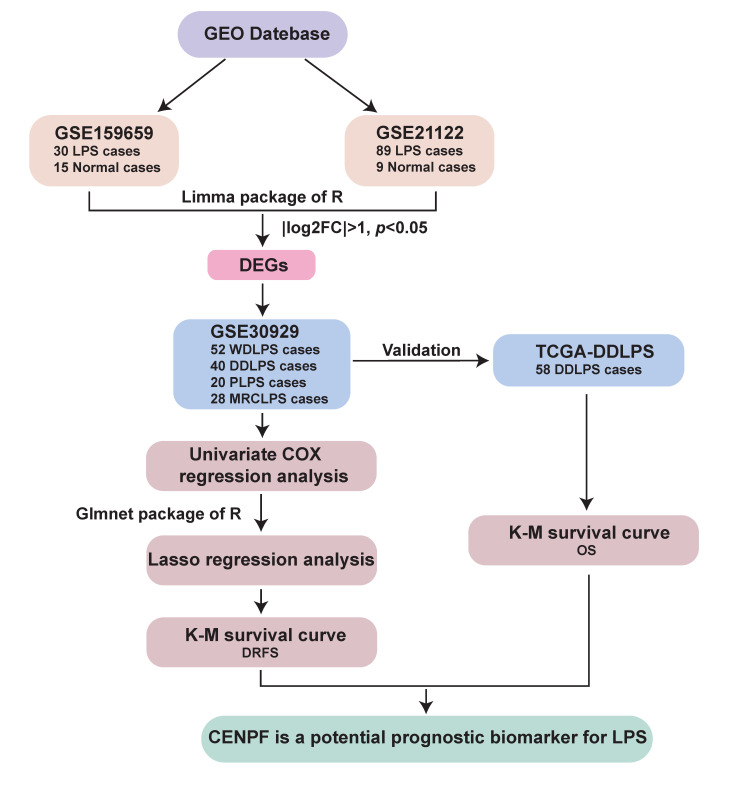
Schematic diagram of candidate genes screening for LPS prognostic biomarkers.

**Figure 2:**
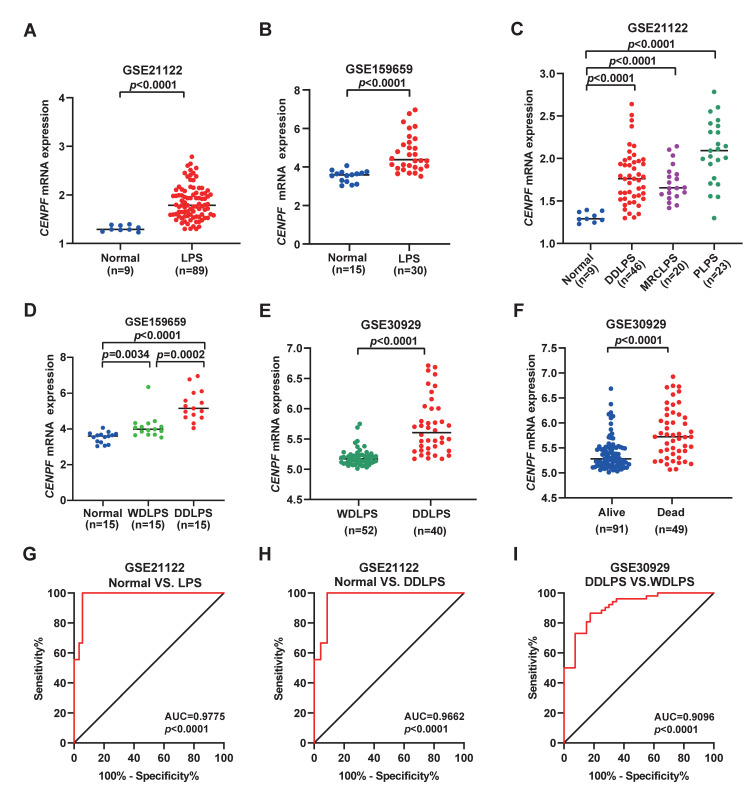
Expression features of *CENPF* in LPS. (**A**–**D**) Compared to normal tissues, *CENPF* expression was upregulated in the LPS of different histological types, including WDLPS, DDLPS, MRCLPS and PLPS. (**E**) Comparison of *CENPF* expression between WDLPS and DDLPS. (**F**) Comparison of *CENPF* expression in LPS patients with different survival status. (**G**,**H**) Sensitivity and specificity of ROC curves of *CENPF* expression in discriminating LPS or DDLPS from normal adipose tissue. (**I**) The discrimination ability of *CENPF* expression between WDLPS and DDLPS. LPS: liposarcoma; DDLPS: dedifferentiated liposarcoma; MRCLPS: myxoid (round cell) liposarcoma; PLPS: pleomorphic liposarcoma; WDLPS: well-differentiated liposarcoma.

As described in ‘Methods’ section, after the screening of DEGs, cell cycle-related *CENPF* was selected as a candidate gene for predicting LPS survival (Figure 1). To explore the potential prognostic value of *CENPF* for LPS, a series of prognostic analyses was performed. Firstly, *CENPF* expression was compared between tumor and normal tissues. As a result, *CENPF* expression was significantly higher in LPS tissue compared to the adjacent non-tumor control (adipose tissue) (*p* < 0.0001, Figure 2A,B). Furthermore, a comparison of normal tissues with different LPS subtypes revealed the significantly elevated *CENPF* expression in WDLPS, DDLPS, MRCLPS and PLPS (*p* < 0.0001, *p* = 0.0034, Figure 2C,D). In comparison with WDLPS patients, patients suffering from DDLPS presented significantly higher *CENPF* expression (*p* = 0.0002, *p* < 0.0001, Figure 2D,E). In addition, *CENPF* expression was significantly higher in the deceased cases than in the living cases (*p* < 0.0001, Figure 2F).

Then, ROC curves of *CENPF* were plotted for discriminating different subgroups. As shown, *CENPF* expression has an outstanding ability of making a distinction between normal fat and LPS (AUC = 0.9775, *p* < 0.0001, Figure 2G) or DDLPS tissues (AUC = 0.9662, *p* < 0.0001, Figure 2H), as well as discriminating DDLPS from WDLPS (AUC = 0.9096, *p* < 0.0001, Figure 2I), indicating that *CENPF* expression level is of diagnostic significance for LPS.

By analyzing the *CENPF* CNAs of LPS patients, the relationship between *CENPF* expression and CNAs was observed: *CENPF* expression in patients with a single copy deletion (n = 20) significantly decreased (*p* < 0.001) compared with diploid normal copy (n = 35) cases (Figure S1).

### 2.2. Overexpression of CENPF Predicts Poor Prognosis of LPS Patients

**Figure 3:**
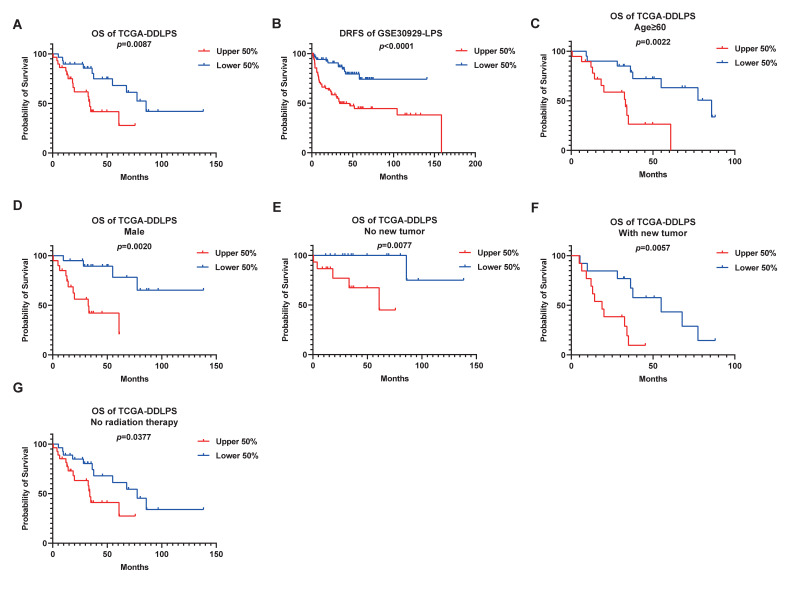
Validation of predictive performances of *CENPF* for LPS by plotting Kaplan–Meier (KM) curves. (**A**) KM curves of OS for DDLPS patients classified by *CENPF* expression (data derived from TCGA dataset). (**B**) Impacts of *CENPF* expression on DRFS. Survival curves based on *CENPF* expression of patients suffering from LPS (data derived from GSE30929 dataset). Verification of prognostic ability of *CENPF* for DDLPS patients classified by different clinicopathological features: (**C**) age: ≥60; (**D**) gender: male; (**E**,**F**) new tumor event after initial treatment; (**G**) radiation therapy. LPS: liposarcoma; DDLPS: dedifferentiated liposarcoma; OS: overall survival; DRFS: distant recurrence-free survival.

To validate the prognostic ability of *CENPF*, Kaplan–Meier curves were plotted to describe the relationship between *CENPF* expression and the OS of LPS patients. In particular, DDLPS patients with high *CENPF* expression were significantly associated with poor OS (*p* = 0.0087) (Figure 3A). Furthermore, survival curves of DRFS demonstrated that high *CENPF* expression predicted the unfavorable DRFS of LPS patients (*p* < 0.0001, Figure 3B). Then, KM curves of OS were performed to investigate the influences of clinical factors on the prognosis of LPS patients with different expression levels of *CENPF*. As illustrated, high *CENPF* expression was associated with poor OS in DDLPS patients who are older than 60 years (*p* = 0.0022, Figure 3C) or male (*p* = 0.0020, Figure 3D). *CENPF* also has a prognostic value in patients with or without a new tumor or not undergoing radiation therapy (*p* = 0.0077, Figure 3E; *p* = 0.0057, Figure 3F; *p* = 0.0377, Figure 3G).

In short, these results suggest that high expression of *CENPF* is associated with a worse prognosis for LPS patients.

### 2.3. Verification of the Prognostic Value of CENPF for LPS Patients by Statistical Analysis

The risk assessment capacity of *CENPF* for LPS prognosis was evaluated through univariate and multivariate Cox regression analyses (Table 1). Univariate analysis showed that *CENPF* expression (*p* = 0.012, HR = 3.112, 95%CI [1.280–7.565]), new tumor status after treatment (*p* = 0.001, HR = 4.331, 95%CI [1.800–10.421]) and cancer status (*p* = 0.010, HR = 4.987, 95%CI [1.479–16.814]) were significantly associated with the OS of LPS patients. Furthermore, multivariate analysis results indicated that *CENPF* expression (*p* = 0.008, HR = 3.752, 95%CI [1.410–9.984]) and new tumor status after treatment (*p* = 0.023, HR = 4.886, 95%CI [1.249–19.120]) could be regarded as independent prognostic factors for LPS patients. To further explore the correlation between *CENPF* and these clinical factors, the differences of *CENPF* expression in LPS patients with different clinical characteristics were analyzed using the chi-square test, but no significant correlation was observed (Table S1).

**Figure 4:**
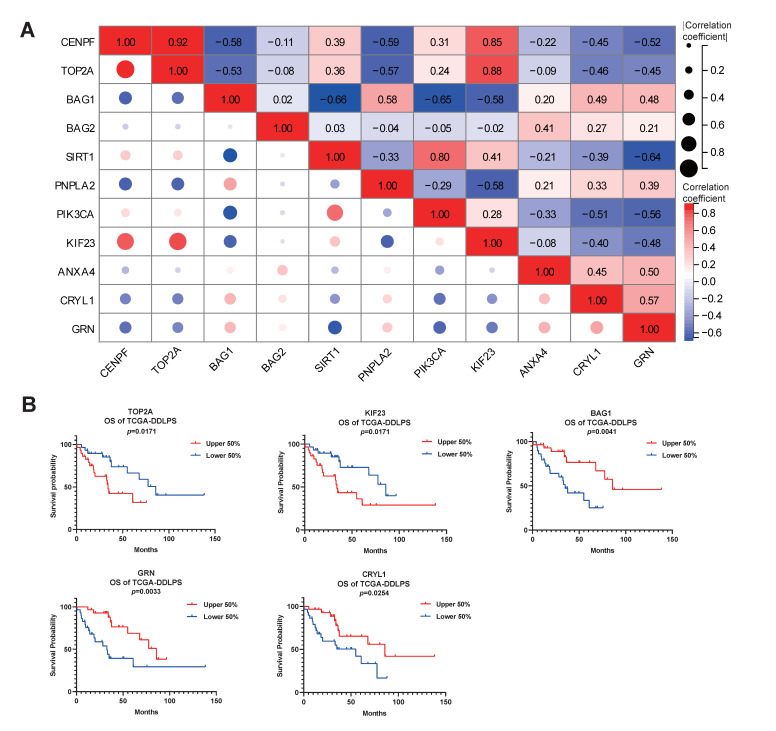
Correlation analysis between *CENPF* and previously reported DEGs or biomarkers of LPS. (**A**) Correlation matrix was plotted by Pearson’s rank correlation test with circles representative of the significance of the correlation. Negative and positive correlation were indicated by blue and red boxes, respectively. Color intensity and absolute value of correlation coefficient were directly proportional to the correlation intensity. (**B**) Validation of the prognostic values of known DEGs or biomarkers of LPS relevant to CENPF (|correlation coefficient| > 0.4) by survival curves. OS: overall survival; DDLPS: dedifferentiated liposarcoma; DEGs: differentially expressed genes.

Next, correlation analysis was performed to evaluate whether the prognostic significance of *CENPF* is correlated with previously reported DEGs or biomarkers of LPS [20,21,22,23]. As the correlation matrix illustrated, *CENPF* was positively correlated with *TOP2A* or *KIF23* (Figure 4A), the high expression of which predicted adverse survival of DDLPS patients (Figure 4B). In contrast, *CENPF* was negatively correlated with *BAG1, CRYL1* and *GRN* (Figure 4A), the high expression of which predicted beneficial survival of DDLPS patients (Figure 4B) indicating that these *CENPF*-related genes did influence the prognosis of LPS patients. In addition, these *CENPF*-related genes exhibited the expression differences between normal and tumor tissues: the expression of *TOP2A* or *KIF23* was significantly upregulated in LPS, while that of *BAG1,* *PNPLA2*, *CRYL1* or *GRN* was significantly downregulated in LPS or MRCLPS compared to normal tissues (Figure S2). In short, these results further verified that CENPF is crucial to the prognosis of LPS patients.

### 2.4. Elucidation of the Potential Functions and Molecular Mechanisms of CENPF in LPS Development

**Figure 5:**
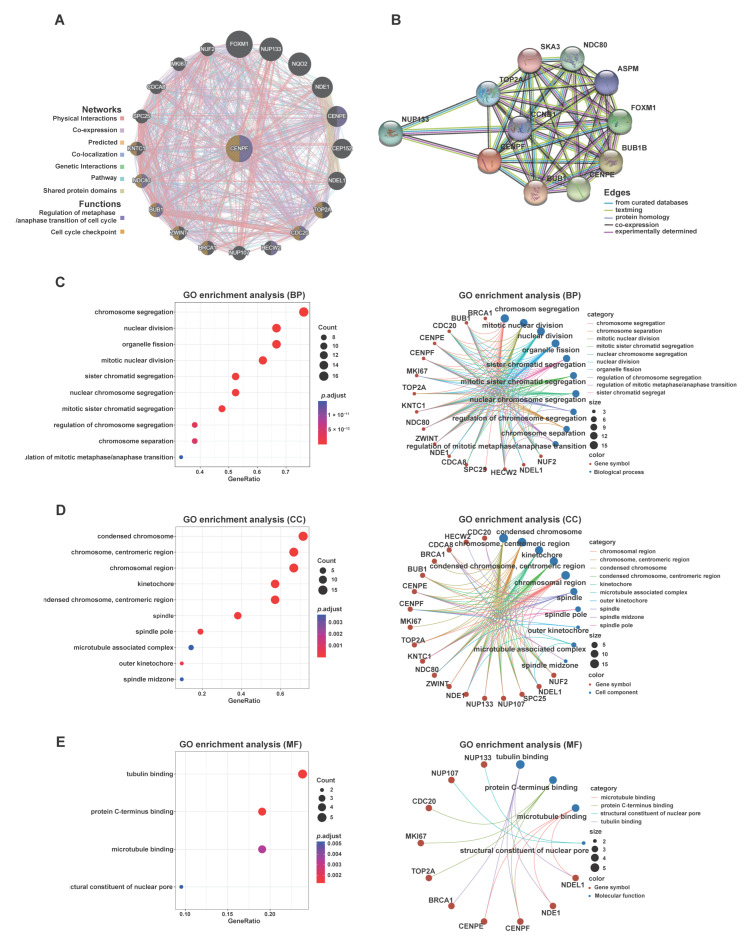
Gene ontology (GO) enrichment analysis of co-expressed genes of *CENPF*. (**A**) Co-expressed gene network of *CENPF*. (**B**) Protein–protein interaction (PPI) network of CENPF. The colors of lines displayed on the networks corresponded to the different interactions with CENPF. Enrichment map and gene-concept network of (**C**) biological process (BP), (**D**) cell component (CC) and (**E**) molecular function (MF). Each dot on the graph represents a group of genes, and the dot size denotes the number of these genes. A redder color indicates a more significant enrichment.

**Figure 6:**
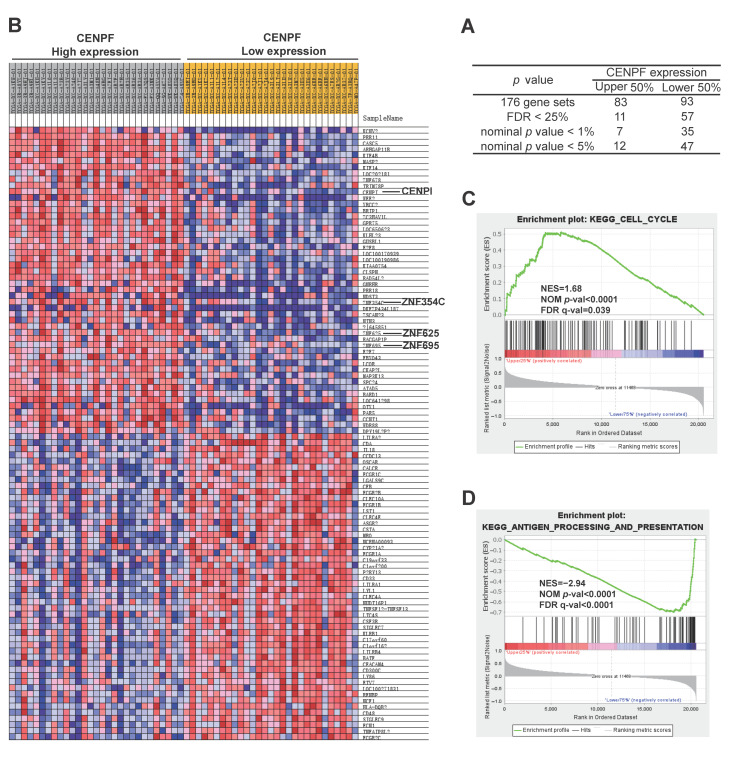
Gene set enrichment analysis (GSEA) of *CENPF*. (**A**) A summary of GSEA analysis. Patients were classified into two subgroups according to *CENPF* expression (upper 50% vs. lower 50%). (**B**) Heat map of top 50 DEGs enriched in *CENPF* high-expression and low-expression subgroup. (**C**,**D**) Gene set enriched in *CENPF* high- and low-expression subgroup, respectively.

To further explore the oncogenic functions of *CENPF* in LPS, GeneMANIA and STRING databases were applied to construct an interaction network of co-expressed genes or the PPI of *CENPF*. As a result, *CENPF* was co-expressed with genes involved in the regulation of cell cycle transition and checkpoint, including *CENPE*, *CDC20*, *TOP2A*, *HECW2*, *BUB1*, *BRCA1*, *ZWINT*, *KNTC1* and *NDC80* (Figure 5A). Additionally, *MKI67* (encoding Ki-67) was most closely related to *CENPF* (Figure 5A). As is known, Ki-67, a classical marker of tumor cell proliferation, is involved in the regulation of cell cycle progression [24]. Significantly, a correlation curve between *MKI67* and *CENPF* was plotted to display a robust correlation (r = 0.9028, *p* < 0.001, Figure S3A). Moreover, the PPI network revealed the association scores of the top five proteins interacting with CENPF: BUB1 (score = 0.998), BUB1B (score = 0.997), CENPE (score = 0.995), FOXM1 (score = 0.992) and NDC80 (score = 0.991) (Figure 5B). Notably, as co-expressed genes (Figure 5A), TOP2A, BUB1 and CENPE also interacted with the CENPF protein (Figure 5B), the high expression of which predicted poor prognosis in cancers [20,25,26]. As shown, a significant correlation between *CENPF* and *TOP2A* was observed (r = 0.9171, *p* < 0.001, Figure S3B). In addition, *CENPF* also revealed a strong relationship with *FOXM1* (r = 0.7397, *p* < 0.001, Figure S3C), which directly regulates *CENPF* [27] to contribute to the progression of mitosis [28]. Moreover, the correlation curves also demonstrated a significant association between *CENPF* and *BUB1* (r = 0.8779, *p* < 0.001, Figure S3D), *NDC80* (r = 0.6653, *p* < 0.001, Figure S3E) or *CDC20* (r = 0.6687, *p* < 0.001, Figure S3F). In addition, *CENPE*, a member of CENPs family, also presented a significant positive correlation with *CENPF* (Figure S4A) and an increased expression in LPS tissues (Figure S4B). Like *CENPF*, the elevated expression of *CENPE* indicated adverse OS or DRFS for LPS patients (Figure S4C,D). Based on these results, GO functional enrichment analyses were performed by Hiplot using the data derived from the co-expressed genes of *CENPF*. In terms of BP, the mostly enriched functional items were “chromosome segregation”, “nuclear division”, “organelle fission”, etc. (Figure 5C). The results of CC suggested that *CENPF* was mainly located in the kinetochore or centromeric region of the condensed chromosome (Figure 5D). As described, *CENPF* assembles to the fibrous corona beyond the outer kinetochore [29]. The functional items of MF were mostly clustered in “tubulin binding”, “protein C-terminus binding” and “microtubule binding” (Figure 5E). Among these, “protein C-terminus binding” is closely related to *CENPF* farnesylation [30], which is essential for the oncogenic function of CENPF. To further elucidate the function and mechanism of *CENPF* exerting on LPS tumorigenesis and progression, GSEA analysis was performed using the data derived from two subgroups of DDLPS patients classified according to *CENPF* expression (high vs. low). Among them, 83 and 93 gene sets were enriched in *CENPF* high (named *CENPF***^high^**) and low (named *CENPF***^low^**) expression subgroups, respectively (Figure 6A). As the heat map illustrated, the expression of ZNFs (zinc finger proteins) increased in the *CENPF***^high^** group (Figure 6B). Likewise, CENPI, a member of CENPs family, also overexpressed in *CENPF***^high^** (Figure 6B), which plays an important role in the localization of CENPF to kinetochores [31]. Like *CENPE* (Figure S4A–D), *CENPI* was significantly correlated with *CENPF*, overexpressed in LPS tissues and anticipated worse OS or DRFS for LPS patients (Figure S4E–H) indicating that these CENPs (CENPF, CENPE and CENPI) are closely related and collaboratively function to exert synergistic effects on mitosis and the cell cycle. Accordingly, gene set “cell cycle” was significantly enriched in *CENPF***^high^** (NES = 1.68, NOM *p*-value < 0.0001, FDR q-value = 0.039, Figure 6C), consistent with GO enrichment analysis results (Figure 5). Gene sets were enriched in *CENPF***^low^,** including “antigen processing and presentation” (Figure 6D).

In short, these results suggested that the potential influences of *CENPF* on LPS progression may involve *CENPF*-mediated regulation of the cell cycle, thereby promoting tumor cell proliferation.

### 2.5. The Correlation between CENPF Expression and Immune Infiltration Level in LPS

**Figure 7:**
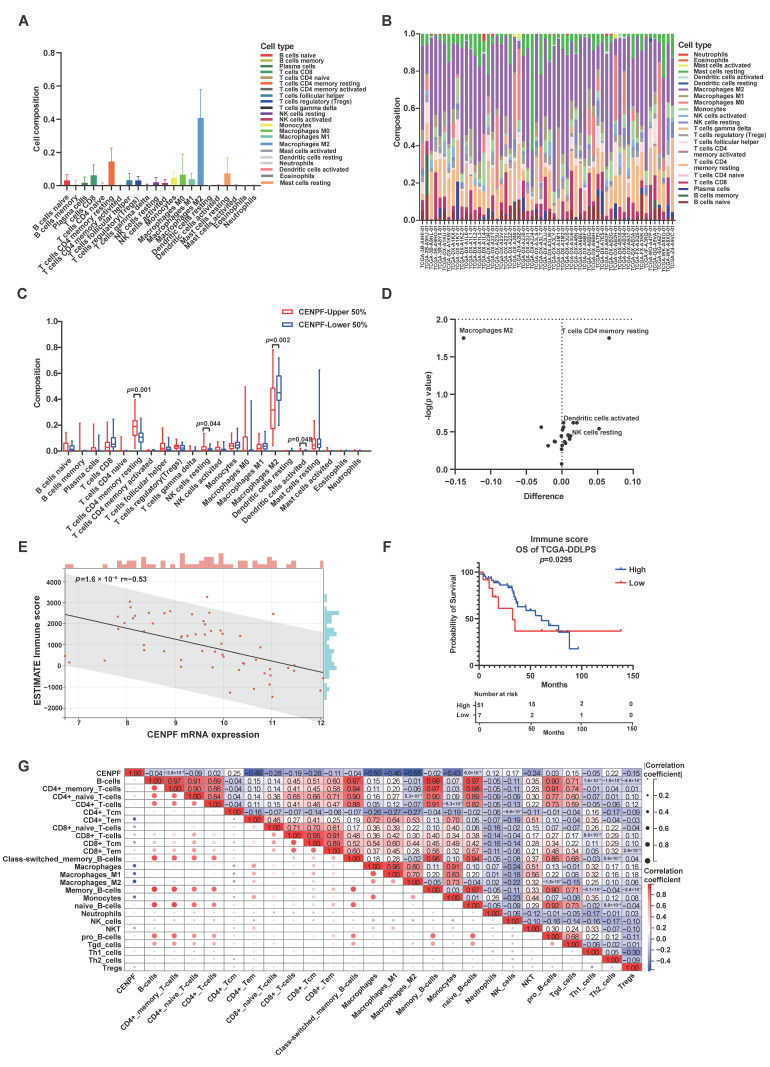
CENPF-related immune infiltration analysis of LPS tumor microenvironment (TME). Composition analysis of immune cells infiltrated in LPS tissues: (**A**,**B**) the proportion of various infiltrating immune cells in TME of LPS. (**C**,**D**) Comparison of the abundance of infiltrating immune cells between *CENPF* high-expression and low-expression subgroup. Analysis of immune score in TME of LPS tissues: (**E**) the correlation between *CENPF* expression and immune score. (**F**) KM curve of OS for DDLPS patients categorized by the immune score (high vs. low) with the number of censored patients at each timepoint (numbers at risk) listed under it. The optimal cut-off of survival curves was determined by X-tile [34]. (**G**) Correlation analysis between *CENPF* expression and scores of immune cells with correlation coefficient marked on the matrix plot. Positive and negative correlations were indicated by red and blue boxes, respectively. Color intensity and absolute value of correlation coefficient were directly proportional to the correlation strength. OS: overall survival; DDLPS: dedifferentiated liposarcoma.

As is known, immune infiltration and the tumor microenvironment (TME) exert significant influences on tumor progression and prognosis [32,33]. To estimate the relationship between *CENPF* and the TME of LPS, a series of comprehensive analyses of immune infiltration were conducted. Firstly, the composition of 22 immune cell subtypes in LPS TME was estimated and visualized by using CIBERSORT. The proportion of ‘T cells CD4 memory resting’ or ‘M2 macrophages’ was high in LPS samples (Figure 7A,B). Then, the differences in immune cell proportion were analyzed between *CENPF*^high^ and *CENPF*^low^ groups, in particular, the proportion of ‘T cells CD4 memory resting’ was significantly higher in *CENPF*^high^ group (Figure 7C,D), which was significantly associated with the OS of LPS, that is, abundant CD4+ T cells (memory resting) might predict an unfavorable prognosis in LPS patients (*p* = 0.0145, Figure S5A). To further elucidate the effects of *CENPF* on the immune infiltration of LPS, the immune scores of LPS cases were assessed by ESTIMTE and xCell. As illustrated, *CENPF* expression was significantly negatively correlated with the immune score of LPS (r = −0.53, *p* = 1.6 × 10^−5^, Figure 7E). The influence of the immune score on LPS prognosis was further interpreted by plotting the survival curve, and the graph suggested that LPS patients with lower immune score exhibited worse OS (*p* = 0.0295, Figure 7F). As the correlation heatmap shows, some kinds of immune cells in LPS TME might be relevant to *CENPF* expression, in particular, the immune score of ‘CD4+ Tem’, ‘macrophages’, ‘M1 macrophages’, or ‘monocytes’ was significantly negatively associated with *CENPF* expression (Figure 7G). Furthermore, the immune score of macrophages or M1 macrophages was significantly associated with the OS of LPS, that is, the higher immune score, the better the prognosis of LPS patients (*p* = 0.026; *p* = 0.044, Figure S5B,C).

Then, the relationship between *CENPF* expression and the infiltration levels of immune cells was evaluated in sarcoma. According to TIMER, the expression level of *CENPF* was positively correlated with tumor purity or the infiltration level of B cells, while negatively correlated with the infiltration level of CD4+ T cells or macrophages in sarcomas (Figure S6).

In short, these findings demonstrated that *CENPF* might be related to immune infiltration and might exert effects on the TME of LPS.

### 2.6. Therapeutic Drugs for Liposarcoma Patients

**Figure 8:**
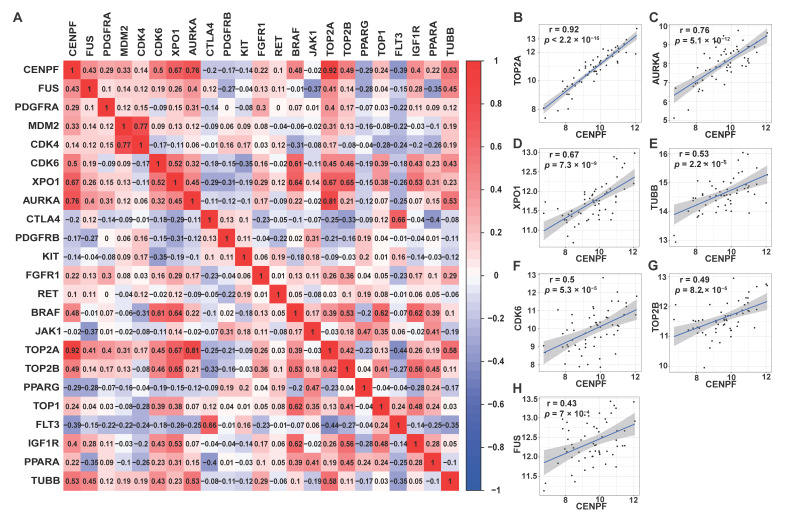
Correlation analysis between *CENPF* and previously identified therapeutic targets for LPS. (**A**) Correlation matrix diagram. (**B**–**H**) *CENPF* expression was significantly positively correlated with several previously identified therapeutic targets for LPS clinical treatment research.

**Figure 9:**
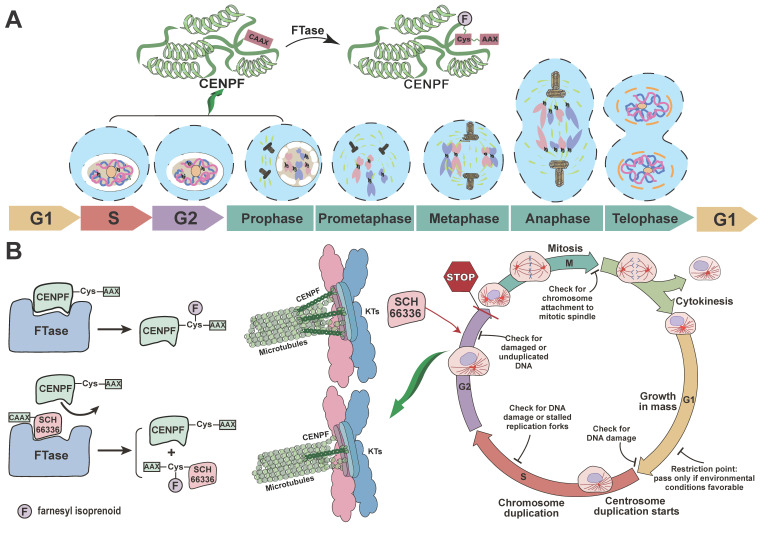
Farnesylation of CENPF affects cell cycle progression. (**A**) Localization of CENPF at different phases of cell cycle. Farnesylation of CENPF occurs from S phase to prophase of mitosis, involving connection of farnesyl isoprene-like compounds to the cysteine thiol group of CAAX peptide motif, catalyzed by farnesyltransferase (FTase). (**B**) SCH66336 (lonafarnib) competitively inhibits the binding of FTase to CAAX peptide of CENPF, thereby repressing its farnesylation. This competitive inhibition leads to a significant reduction of active CENPF in nucleus at prophase of mitosis and early centromere. As a result, G2/M transition is delayed due to the dysfunction of CENPF, thus cell cycle progression is restrained. F: farnesyl isoprenoid; FTase: farnesyl transferase; KTs: kinetochores; CAAX: CAAX box peptide (C: cysteine; A: an aliphatic amino acid; X: methionine, threonine, serine or glutamine); SCH66336: also known as Lonafarnib, a FTase inhibitor.

It has been reported that in phase II clinical trials, the Progress Free Interval (PFI) is considered as a primary end point and suggests the necessity of second-line treatment if PFI >40% at 12 weeks [35]. In our study, the survival curve was plotted using PFI data of LPS patients who received drug treatment, which revealed that probability of survival was greater than 40% at three months (Figure S7). Although the above data may be not derived from phase II clinical trials, the result might in part reflect the effect of the drugs applied for LPS treatment. However, determining how to effectively treat LPS patients is always a challenge. Thus, a series of previously reported therapeutic drugs for LPS patients were summarized in Table S2, which provided integrated information of therapeutic drugs for LPS clinical application, including name, targets, dosage, clinical trial stage and mechanisms. As summarized, several drugs have been already marketed or are in phase III clinical trials (Table S2). According to the mechanisms of action, several drugs were related to cell cycle arrest and microtubule binding (Table S2), for instance, trabectedin, eribulin, doxorubicin, epirubicin, abemaciclib, palbociclib, ribociclib, plocabulin, docetaxel, etc. [36,37,38]. In the present study, CENPF, a regulator of the cell cycle and microtubule attachment, was identified as a potential prognostic biomarker and prospective therapeutic target for LPS. To probe the possibility of its application in LPS treatment, Pearson’s correlation analysis of CENPF with these previously identified therapeutic targets (Table S2) for LPS was performed (Figure 8). As a matrix illustrated, *CENPF* was significantly positively associated with *FUS* (r = 0.43, *p* = 7 × 10^−4^), *CDK6* (r = 0.5, *p* = 5.3 × 10^−5^), *XPO1* (r = 0.67, *p* = 7.3 × 10^−9^), *AURKA* (r = 0.76, *p* = 5.1 × 10^−12^), *TOP2A* (r = 0.92, *p* < 2.2 × 10^−16^), *TOP2B* (r = 0.49, *p* = 8.2 × 10^−5^) and *TUBB* (r = 0.53, *p* = 2.2 × 10^−5^) (Figure 8A), further confirmed by correlation analysis between two genes (Figure 8B–H). In addition, the potential association of CENPF and its co-expressed genes with therapeutic drug sensitivity in sarcoma was estimated by drug sensitivity analysis through GSCALite platform. The graph revealed that CENPF and its co-expressed genes displayed a significant association with drug sensitivity of Trametinib, selumetinib and RDEA119 for sarcoma treatment (Figure S8). To further explore the possibility of its utilization in LPS treatment, lonafarnib (SCH66336), a small molecule inhibitor of CENPF [39], was investigated (Figure 9). Lonafarnib, as one of the farnesyltransferase inhibitors (FTIs), has been approved by the Food and Drug Administration (FDA) for marketing to treat progeria by restricting progerin farnesylation and cell membrane embedding [40,41]. Likewise, lonafarnib also acts on CENPF to hinder its farnesylation (Figure 9) leading to the dampened protein activity of CENPF and thus the repressed cell proliferation [42,43]. In a prospective view, it is expected that lonafarnib may become a potential molecular-targeted therapeutic drug for LPS by targeting CENPF.

## 3. Discussion

LPS is a type of soft tissue sarcoma that accounts for 20% of the mesenchymal malignancies [44] with a high prevalence in the neck, head, retroperitoneum and extremities [45]. The metastasis of LPS could affect the survival outcome of patients, which is a challenge to the surgical resection and the stable state maintained by chemotherapy [46,47]. In recent years, molecular targeted therapy has been becoming a promising treatment modality that contributes to improving the survival of LPS patients [48]. Thus, it is of great significance to explore potential predictors for the risk assessment of LPS patients, which may convert to the prospective molecular targets. CENPF, a cell cycle-dependent microtubule-related protein, acts as an oncogene in various tumors. As a kinetochore protein, CENPF is an important regulator of mitosis and the cell cycle through controlling microtubule capture and G2/M progression [39,49], but the effect of *CENPF* on the prognosis of LPS patients has not been reported yet, and the molecular mechanisms of their actions in LPS oncogenesis are still unclear.

In the present study, *CENPF* was identified as a potential predictor for LPS survival through screening DEGs between LPS and normal tissue. In detail, *CENPF* expression was significantly higher in LPS than that in normal adipose tissue, and patients afflicted with DDLPS presented significantly higher *CENPF* expression than WDLPS patients. These results suggested that *CENPF* expression was upregulated in tumor tissues, especially the dedifferentiated histologic subtype, indicating the involvement of *CENPF* in liposarcomagenesis and development. In addition, *CENPF* expression showed significant differences in patients with different survival outcomes. The abnormal expression of *CENPF* has been reported [11] in multiple cancers, including prostate cancer [50], HCC [15] and nasopharyngeal carcinoma [51], indicating that high *CENPF* expression is a malignant indicator. Furthermore, Kaplan–Meier plots showed that upregulation of *CENPF* in LPS patients always leads to worse OS and DRFS, especially in patients afflicted with DDLPS (more malignant). As is known, DDLPS has a high recurrence and metastasis rate [52] displaying a strong aggressiveness. This suggests that *CENPF* overexpression may be closely associated with the malignancy of LPS and contribute to the accelerated tumor progression. Univariate and multivariate Cox regression analysis further demonstrated that *CENPF* functions as a risk indicator in promoting progressive disease and could be considered as an independent prognostic biomarker for LPS patients.

Therefore, the molecular mechanism of CENPF-mediated oncogenic progression was of greater concern. The association of *CENPF* with some known DEGs or biomarkers in LPS was explored. *TOP2A* and *KIF23*, significantly and positively associated with *CENPF*, have been reported to be highly expressed in LPS [20,53]. *PNPLA2*, *CRYL1* and *GRN*, exhibiting a negative correlation with *CENPF*, have been reported to be downregulated in MRCLPS [22,53]. *BAG1*, negatively related to *CENPF*, was identified by our previous study as a protective prognostic biomarker for LPS. The expression differences of these DEGs between LPS and normal adipose tissue were confirmed using the data from the GSE21122 dataset. Survival analysis of these DEGs was performed to further verify their influences on LPS prognosis, and the results confirmed that high expression of *CENPF* positively-correlated genes corresponded to worse OS. On the contrary, *CENPF* negatively-correlated genes corresponded to better OS, further verifying that *CENPF* is a malignant indicator.

According to the results of GeneMANIA and STRING, correlation curves of six co-expressed genes (*MKI67*, *TOP2A*, *BUB1*, *NDC80*, *CDC20* and *FOXM1)* displayed significant positive correlations with *CENPF* expression. As previously described, these genes are involved in cell cycle regulation [20,24,27,54,55,56]. It is noteworthy that TOP2A, BUB1 and CENPE are not only co-expressed but also interact with CENPF. TOP2A, encoding topoisomerase IIα, is crucial to maintaining mitotic chromosome condensation [57]; BUB1, a checkpoint kinase, is able to interact with CENPF or CENPE and is essential for the localization of CENPF or CENPE to kinetochores and the chromosome condensation during mitosis [58,59]; CENPE, a kinetochore protein, interacts with BUB1 and binds to microtubules of the spindle playing key roles in mitosis and cell cycle progression [12,42,59]. Thus, our findings indicate that these genes are closely related at both mRNA and protein levels to cooperatively function in mitosis and cell cycle, thereby contributing to the accelerated LPS progression. Further exploration of the GO enrichment analysis revealed that *CENPF* functions in the cell cycle through regulating mitosis and microtubule binding in various cancers, for instance, CENPF contributes to the deterioration of adrenocortical carcinoma by affecting the cell cycle [14]. In the heatmap of the GSEA analysis, several ZNFs were upregulated in the *CENPF*^high^ group, which belong to the zinc finger transcription factor family and have profound impacts on cancer progression [60,61,62,63,64,65,66], implying a presumable connection between CENPF and these ZNFs. Additionally, CENPI, a constitutive protein of kinetochore, was synergistically upregulated with CENPF (as GSEA results shown), which specifies localization of CENPF to kinetochores and is essential for chromosome segregation and mitosis [31]. Like CENPF, CENPI is overexpressed in multiple cancers and deciphers worse prognosis [67,68,69,70,71]. Our findings suggest the possibility of a cooperative regulatory relationship between CENPF and CENPE or CENPI, thus they functionally synergistically promote liposarcomagenesis and progression.

The tumor microenvironment (TME), composed of immune cells, stromal cells, blood vessels and extracellular matrix (ECM), engenders profound influences on tumorigenesis and progression [72,73]. In general, immune cells were categorized into two classes: innate and adaptive immune cells [74]. In immune infiltrated tumors, these immune cells are extensively dispersed throughout tumor tissues with active immune responses, imposing double effects: pro- and anti-tumorigenic [75]. Immune cells in TME are associated with the prognosis and treatment effect of various cancers [73,75], including soft-tissue sarcomas (STS) [76,77]. It is of significance to identify candidate prognostic biomarkers to discern patients inclined to benefit from immunotherapy. In the present research, the relationship of immune cell scores with *CENPF* expression was investigated. As previously described, immune score is predictive of clinical outcomes: patients with high immune scores display longer survival [78,79]. Our findings demonstrated a negative correlation between *CENPF* expression and immune score of LPS TME, and low immune score or high *CENPF* expression forecasted worse survival of LPS patients, implying that *CENPF* might be regarded as a malignant indicator of tumor immune infiltration-related survival. As previously mentioned, the microenvironment rich in effective memory CD4+ T cells (Tem) is correlated with a better prognosis [80], and patients with a richer M1 macrophage infiltration also present a favorable prognosis [76,77]. In our study, *CENPF* was significantly negatively associated with CD4+ Tem or M1 macrophage and deciphered poor survival of LPS patients indicating the underlying connection between CENPF and CD4+ Tem or M1 macrophage, that is, CENPF might counterbalance the impacts of protective immune responses on LPS. LPS patients with high *CENPF* expression might resist anti-tumor immune attack to deteriorate progressively, thus CENPF might be a facilitator of tumor escape (needs to be verified). Therefore, therapeutically targeting CENPF combined with immunotherapy might be an attractive strategy for the treatment of LPS. As the underlying molecular mechanisms of CENPF-mediated liposarcomagenesis and progression are defined, CENPF is expected to become a promising therapeutic target.

Through analysis of association between CENPF and previously identified therapeutic targets, we speculated that LPS patients with a high expression of CENPF might be more sensitive to drugs targeting these CENPF-related targets. Combined application of CENPF antagonists/inhibitors and these targeted drugs might improve responses for LPS to achieve significant clinical benefits. Thus, developing CENPF as a therapeutic target is an attractive strategy for the treatment of LPS. In this case, lonafarnib (SCH66336), a small molecule inhibitor of CENPF approved by FDA [39,40,42], has caught our attention. As is known, CENPF is a significant regulator of mitosis and the cell cycle, and its level varies in different stages of the cell cycle [13]. Farnesylation modification of CENPF, occurring between the S phase and prophase of mitosis, is essential for the G2/M transition to maintain normal progression of the cell cycle [49,81]. SCH66336, targeting farnesylation of CENPF, activates the G2/M checkpoint and leads to G2/M arrest [42]. Looking forward, it is expected that lonafarnib (SCH66336) may become a potential molecular targeted therapeutic drug for LPS treatment by targeting CENPF, or combined with other drugs, to improve the clinical response and survival time of LPS patients.

## 4. Materials and Methods

### 4.1. Data Collection and Interpretation

Gene expression profiling and clinical data of LPS patients were retrieved from The Cancer Genome Atlas (TCGA) and Gene Expression Omnibus (GEO) databases. Clinicopathological data derived from TCGA dataset includes gender, treatment outcome, tumor size, DNA copy number alterations (CNAs), survival status and time information of 59 DDLPS cases. GEO datasets: GSE159659 includes 30 LPS and 15 normal samples; GSE21122 contains 89 LPS and 9 normal samples; GSE30929 is composed of 140 LPS samples and provided survival status and time data of patients without distant metastasis recurrence (DRFS: distant recurrence-free survival).

### 4.2. Screening of Gene with Potential Prognostic Value for LPS

The flowchart of candidate gene screening was illustrated in Figure 1. Differentially expressed genes (DEGs) between LPS and normal tissues were obtained by using Limma package based on R language from GSE159659 and GSE21122 dataset, respectively. DEGs were defined as representative differences with |log2FC| >1, *p* < 0.05. The overlapped genes derived from these two groups of DEGs were further filtered by regression models using the data from GSE30929 dataset. First, SPSS was applied to conduct univariate Cox regression analysis, and genes with *p* < 0.05 were included. Then the Glmnet package was used to carry on lasso regression analysis based on R language. Finally, survival curves were used to validate the prognostic values of candidate genes. After filtering, *CENPF* was selected as a potential prognostic biomarker for LPS.

### 4.3. Comparison of the Relationship between CENPF Expression and Clinical Features

To depict the expression features of *CENPF* in LPS, patients were categorized into subgroups according to their clinical features, and the differences of *CENPF* expression were shown in scatter plots. After that, receiver operating characteristic (ROC) curves were conducted to estimate the discrimination ability of *CENPF* expression in making a distinction between LPS and normal tissues or between WDLPS and DDLPS tissues. In addition, CNAs data were divided into three categories (copy number amplification, diploid normal copy and single copy deletion) to investigate whether *CENPF* expression is relevant to DNA CNAs. All of the above analyses were plotted by using GraphPad Prism 8.4.3, and it can be considered as a significant difference if *p* < 0.05.

### 4.4. Validation of the Prognostic Value of CENPF for LPS

To estimate the prognostic ability of *CENPF* in LPS, Kaplan–Meier (KM) curves of Overall Survival (OS) were plotted by using clinical data of DDLPS patients derived from TCGA dataset. Additionally, KM curves of DRFS were plotted by using clinical data of LPS patients derived from GSE30929 dataset. Correlation matrix analysis of *CENPF* and previously proposed LPS biomarkers or DEGs was visualized by heat map in Sangerbox 3.0 (http://vip.sangerbox.com/index.html) (accessed on 10 July 2022).

To analyze the association between *CENPF* expression and clinicopathological parameters, chi-square test was conducted by SPSS 25.0 software using expression and clinical data of DDLPS patients. Multivariate Cox regression analysis was applied to evaluate the independent prognostic value of *CENPF* expression by using SPSS 25.0, and *p* < 0.05 was considered to be statistically significant.

### 4.5. Construction of co-Expressed Gene and Protein–Protein Interaction (PPI) Network

Co-expressed genes of *CENPF* were explored using GeneMANIA database (https://genemania.org/) (accessed on 19 June 2022). In addition, a PPI network was constructed to explore interacting proteins of CENPF by means of STRING database (https://string-db.org/) (accessed on 23 April 2022). Furthermore, the correlation between *CENPF* and its co-expressed genes was estimated by Pearson’s correlation test and linear regression analysis using GraphPad.

### 4.6. Functional Enrichment Analysis of CENPF

Hiplot (https://hiplot-academic.com/) (accessed on 11 July 2022) was adopted to annotate gene function for GO enrichment analysis, including molecular function (MF), biological process (BP) and cellular component (CC). Enrichment maps and gene-concept networks were output to exhibit enrichment results. Gene Set Enrichment Analysis (GSEA) was introduced to investigate the function enrichment of *CENPF* and explore the molecular mechanisms underlying *CENPF*-mediated LPS tumorigenesis and progression. In detail, 58 TCGA-DDLPS cases were categorized into two groups according to *CENPF* expression (high vs. low). After running, the output results were visualized as heatmaps, peak maps, ranked genes, etc. Statistical difference thresholds were set as |NES|> 1, NOM *p*-value < 0.05 and FDR q-value < 0.25.

### 4.7. Comprehensive Analysis of Immune Infiltration

CIBERSORT (https://cibersort.stanford.edu/) (accessed on 28 June 2022) [82], was introduced to estimate the abundance of immune cells based on gene expression data from TCGA dataset. LM22, a reference gene expression feature set for 22 immune cell subtypes, was employed to analyze percentage of these immune cell.

ESTIMATE (https://bioinformatics.mdanderson.org/estimate/index.html) (accessed on 29 June 2022) [83] was applied to estimate the ratio of stromal and immune cells to non-tumor components using gene expression data and obtain the immune scores for LPS. The correlation scatter plot was visualized by Sangerbox 3.0 to investigate the relationship between *CENPF* expression and immune scores in LPS.

xCell (https://xcell.ucsf.edu/) (accessed on 29 June 2022) is a method that combines enrichment analysis with a deconvolution approach to convert gene expression profiles into enrichment scores for samples [84]. In Sangerbox 3.0, xCell algorithm was selected to obtain the enrichment scores of 64 cell types (stromal and immune cells) and the immune score of each sample.

The infiltration of six immune cell types (B cells, CD4+ T cells, CD8+ T cells, neutrophils, macrophages and dendritic cells) in sarcoma was estimated by TIMER 2.0 (http://timer.comp-genomics.org/) (accessed on 20 June 2022). Gene modules were used to analyze the correlation between *CENPF* expression and immune infiltration in sarcoma (including LPS). Statistically significant differences: *p* < 0.05.

### 4.8. Drug Sensitivity Analysis of CENPF in Soft Tissue Sarcoma

GSCALite (http://bioinfo.life.hust.edu.cn/web/GSCALite/) (accessed on 1 June 2022) was introduced to conduct drug sensitivity analysis [85]. Based on data of CENPF and its co-expressed genes, “TCGA-SARC” (sarcoma) and “Drug Sensitivity” in GSCALite were chosen and set, and correlation in the Spearman algorithm between gene expression and small molecule/drug sensitivity (IC50) derived from GDSC database were performed.

## 5. Conclusions

In conclusion, CENPF may be considered as a promising biomarker regarding LPS prognosis as well as tumor immune infiltration and may be a molecular therapeutic target for LPS patients.

## Figures and Tables

**Table 1:** Univariate and multivariate analysis of factors associated with LPS survival. The bold number indicates the significance (*p* < 0.05).

Subgroup	Univariate Analysis		Multivariate Analysis	
	Hazard Ratio (95% CI)	*p* Value	Hazard Ratio (95% CI)	*p* Value
All patients (n = 59)				
CENPF expression: high vs. low (n = 58)	3.112 (1.280–7.565)	**0.012**	3.752 (1.410–9.984)	**0.008**
New tumor after treatment: yes vs. no (n = 58)	4.331 (1.800–10.421)	**0.001**	4.886 (1.249–19.120)	**0.023**
Cancer status: with tumor vs. tumor free (n = 56)	4.987 (1.479–16.814)	**0.010**	3.861 (0.763–19.533)	0.102
Gender: female vs. male (n = 59)	1.997 (0.919–4.338)	0.081		
Age: ≥60 vs. < 60 (n = 59)	1.860 (0.700–4.944)	0.213		
Metastasis: yes vs. no (n = 25)	2.218 (0.569–8.646)	0.251		

## Data Availability

Publicly available datasets were analyzed in this study. The datasets analyzed for this study can be found in the TCGA database (https://www.cancer.gov/about-nci/organization/ccg/research/structuralgenomics/tcga) (accessed on 17 January 2022) and GEO database (https://www.ncbi.nlm.nih.gov/gds/?term=) (accessed on 13 November 2021).

## Supplementary Materials

The following supporting information can be downloaded at: https://www.mdpi.com/article/10.3390/ijms24087010/s1.
