# Supplementary material for: Deciphering the Prognostic and Therapeutic Significance of Cell Cycle Regulator CENPF: A Potential Biomarker of Prognosis and Immune Microenvironment for Patients with Liposarcoma
Source: Int J Mol Sci. 2023 Apr 10;24(8):7010. doi: 10.3390/ijms24087010 (PMC10139200; doi:10.3390/ijms24087010)
Supplement: Supplementary file 1 [file ijms-24-07010-s001.zip › ijms-2237968-supplementary.pdf]

Supplementary

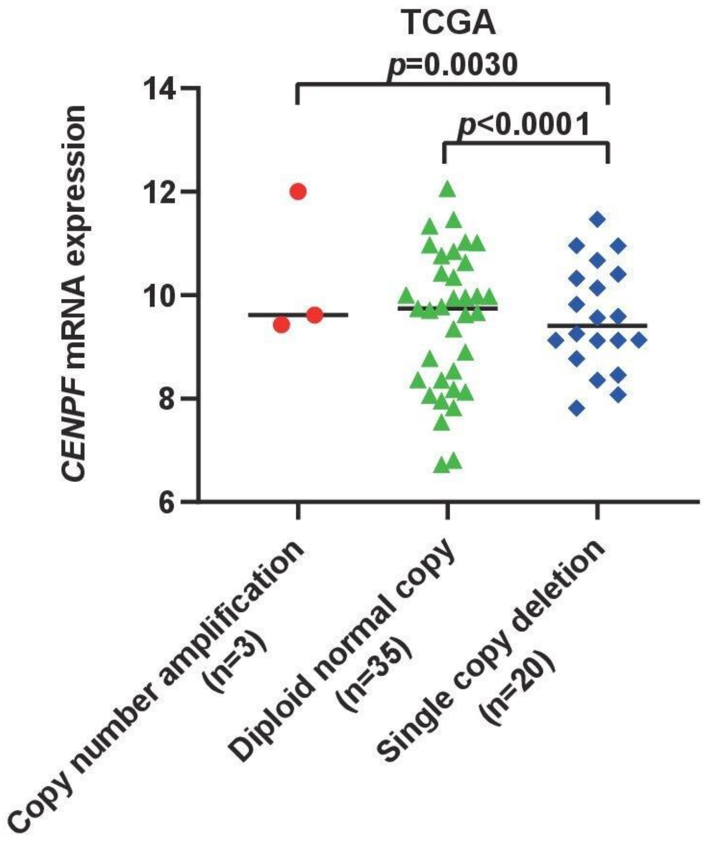

Figure S1. The relationship between mRNA level and DNA copy number alterations (CNAs) of *CENPF* in LPS. Copy number amplification: +1, n= 3; diploid normal copy: 0, n=35; single copy deletion: -1, n= 20.

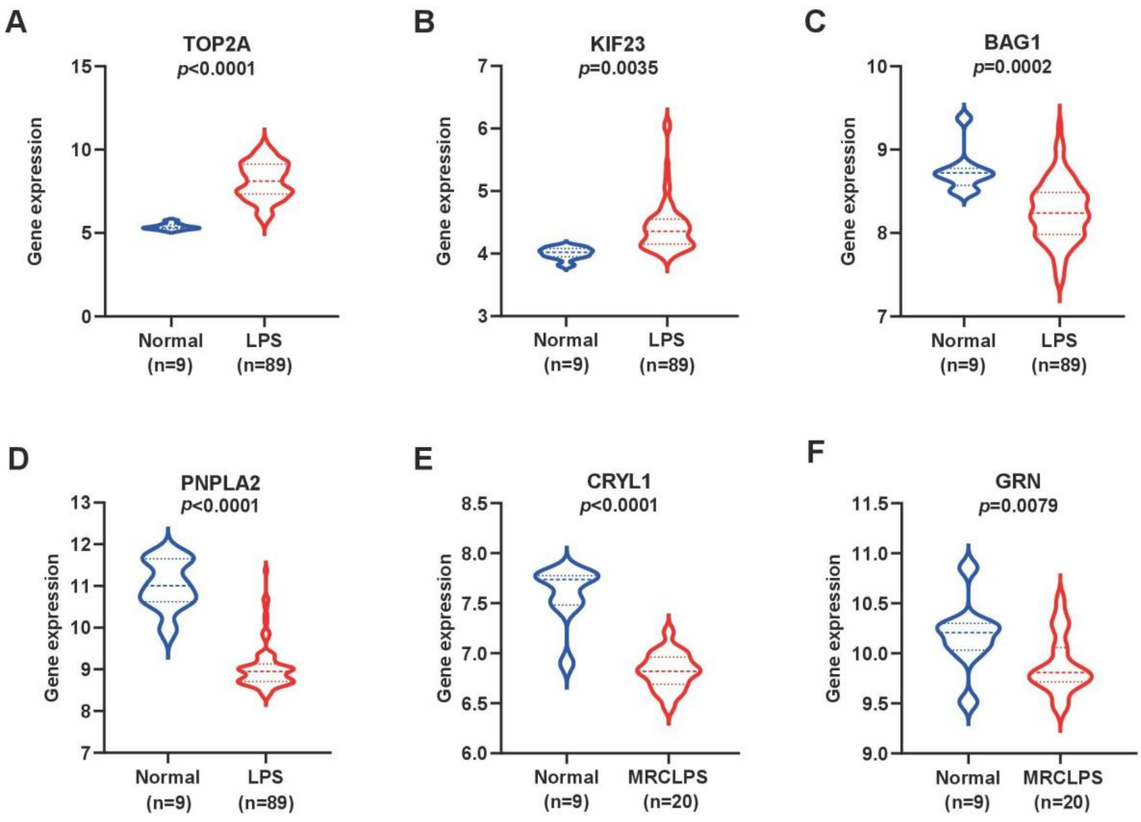

Figure S2. Validation of the expression differences of known biomarkers or DEGs between normal and LPS tissues. (A, B) Comparison of *TOP2A* and *KIF23* expression between normal and LPS tissues, respectively. (C, D, E, F) the expression levels of *BAG1*, *GRN*, *CRYL1* and *PNPLA2* were decreased in LPS or MRCLPS, compared to normal tissues. Expression profile data was derived from GSE21122 dataset. LPS: liposarcoma; MRCLPS: myxoid (round cell) liposarcoma.

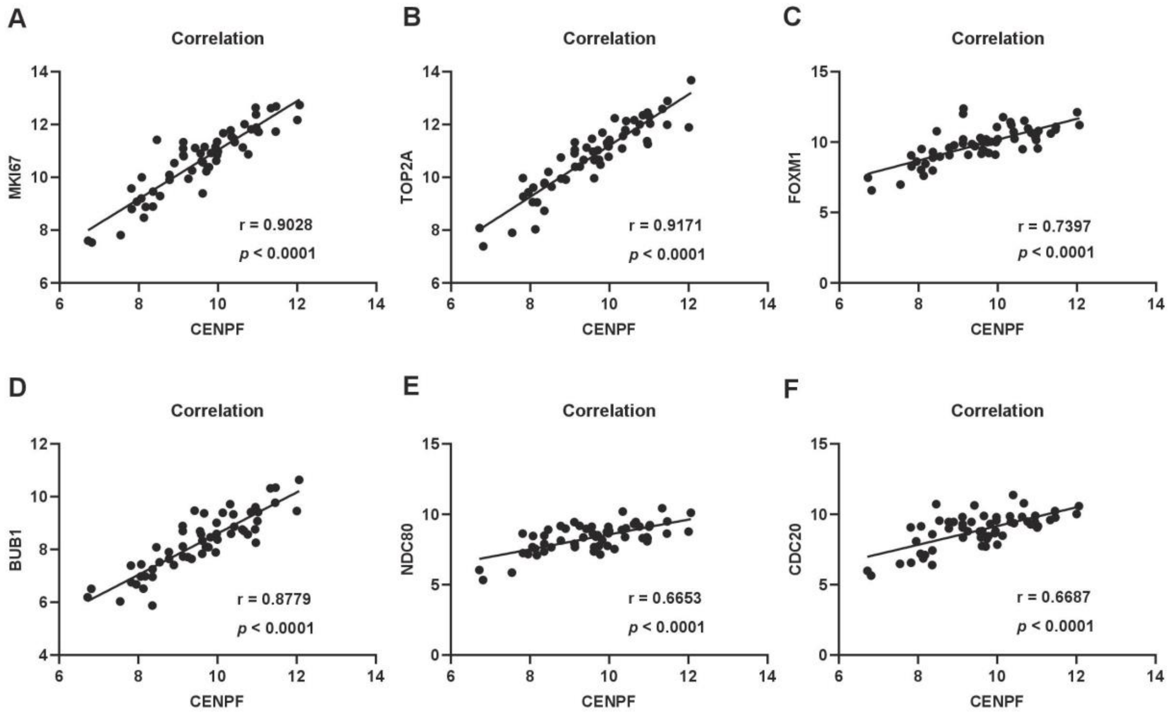

Figure S3. Correlation analysis between *CENPF* and its co-expressed genes derived from GeneMANIA. (A) *CENPF* and *MKI67*. (B) *CENPF* and *TOP2A*. (C) *CENPF* and *FOXM1*. (D) *CENPF* and *BUB1*. (E) *CENPF* and *NDC80*. (F) *CENPF* and *CDC20*.

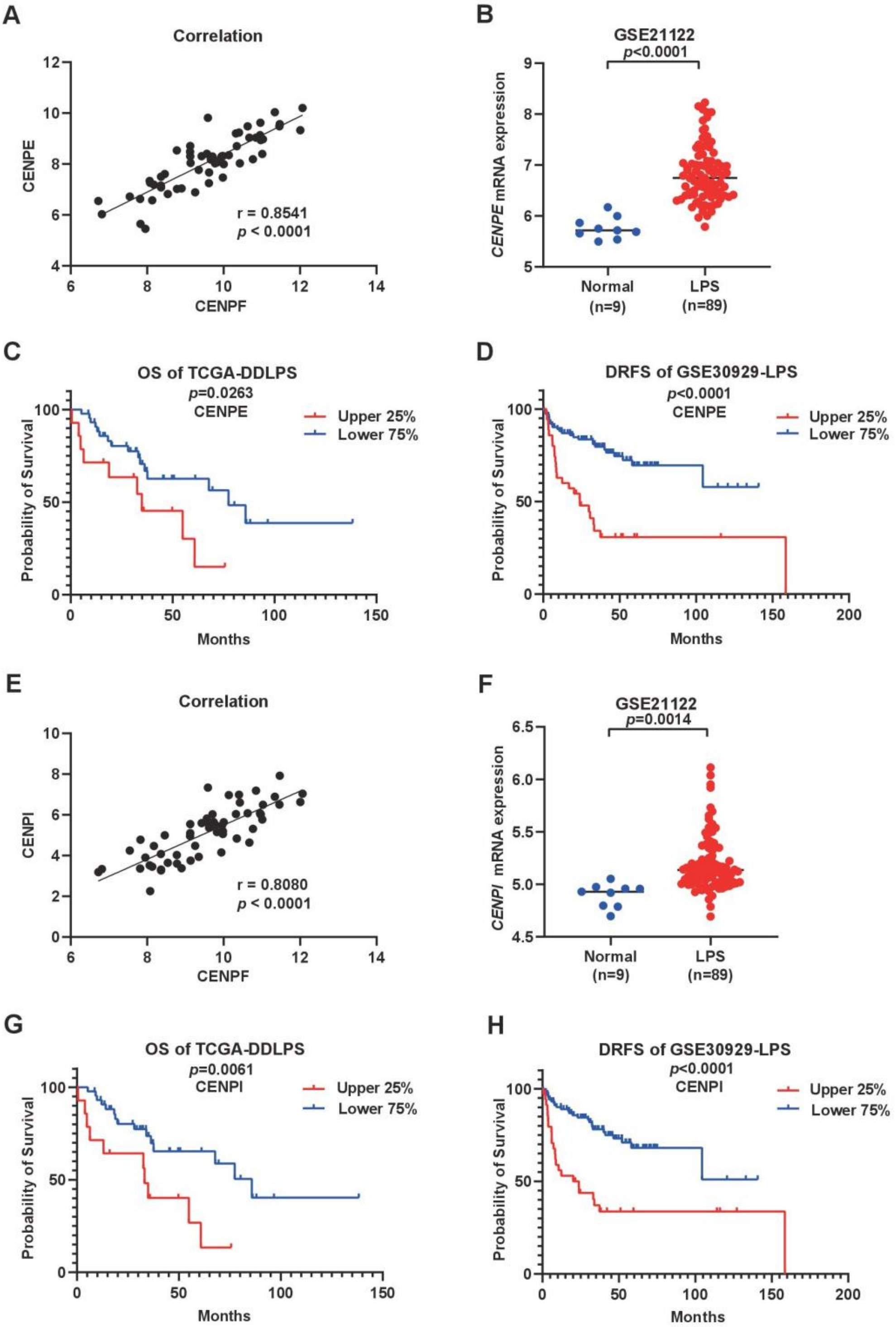

Figure S4. Identification of prognostic value of CENPE or CENPI for LPS patients. (A, E) Correlation analysis between *CENPF* and its partner *CENPE* or *CENPI*. (B, F) Compared to normal tissues, *CENPE* or *CENPI* expression was up-regulated in LPS. (C, D, G, H) High expression of *CENPE* or *CENPI* deciphered a worse OS or DRFS of LPS patients.

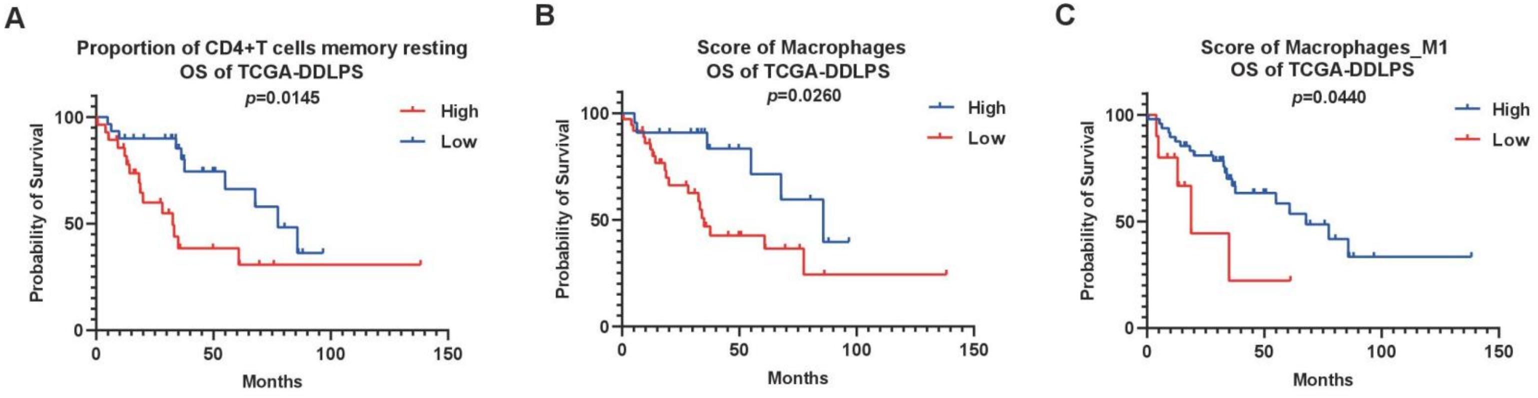

Figure S5. Immune-related overall survival (OS) curves in DDLPS. (A) KM curves depicting the influence of the proportion of CD4+ T cells (memory resting) on OS of DDLPS patients. (B, C) KM curves depicting the impact of the immune score of macrophages and M1 macrophages on OS of DDLPS patients, respectively. OS: overall survival; DDLPS: dedifferentiated liposarcoma. The optimal cut-off of survival curves were determined by X-tile.

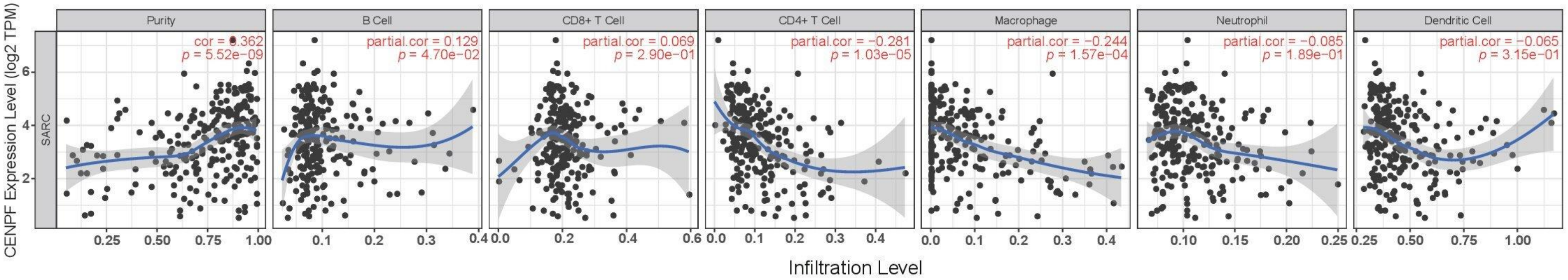

Figure S6. Association of *CENPF* expression and immune infiltration level in sarcoma. The *CENPF* expression has a significant positive correlation with the infiltration level of tumor purity or B cell, while significant negative correlation with the infiltration level of CD4+ T cell or macrophage in sarcoma.

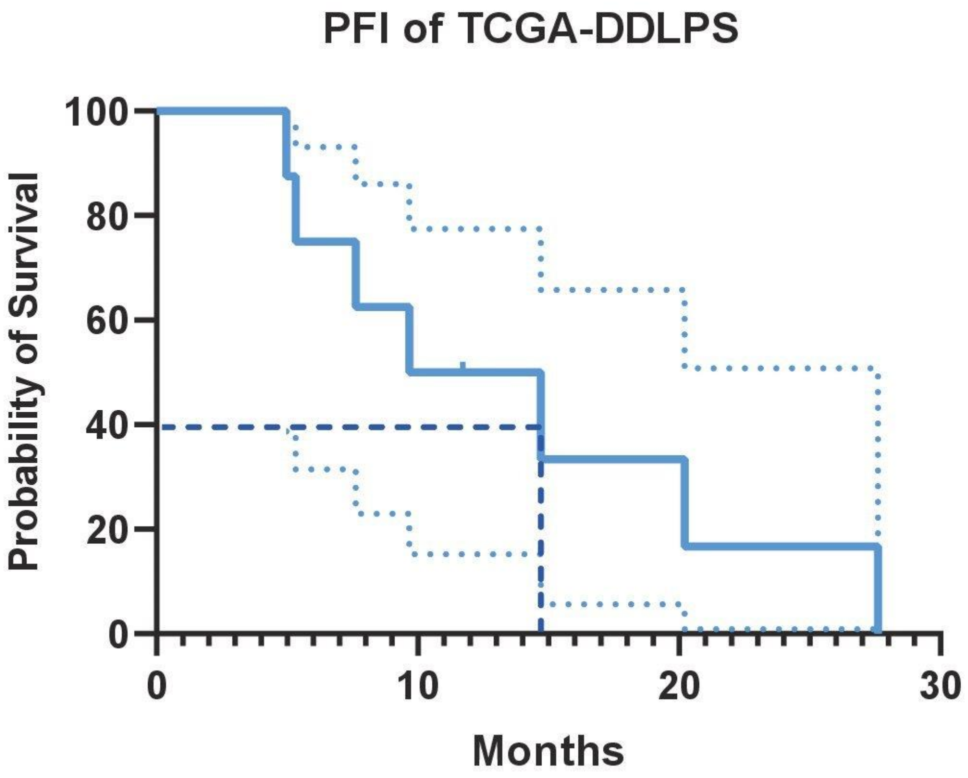

Figure S7. Kaplan-meier (KM) curve of progress free interval (PFI) for DDLPS patients. PFI curve was shown in blue solid line. The light blue dotted lines represent 95%CI, and the dark blue dotted line represents the survival time with a 40% probability of survival. DDLPS: dedifferentiated liposarcoma; PFI: progress free interval.

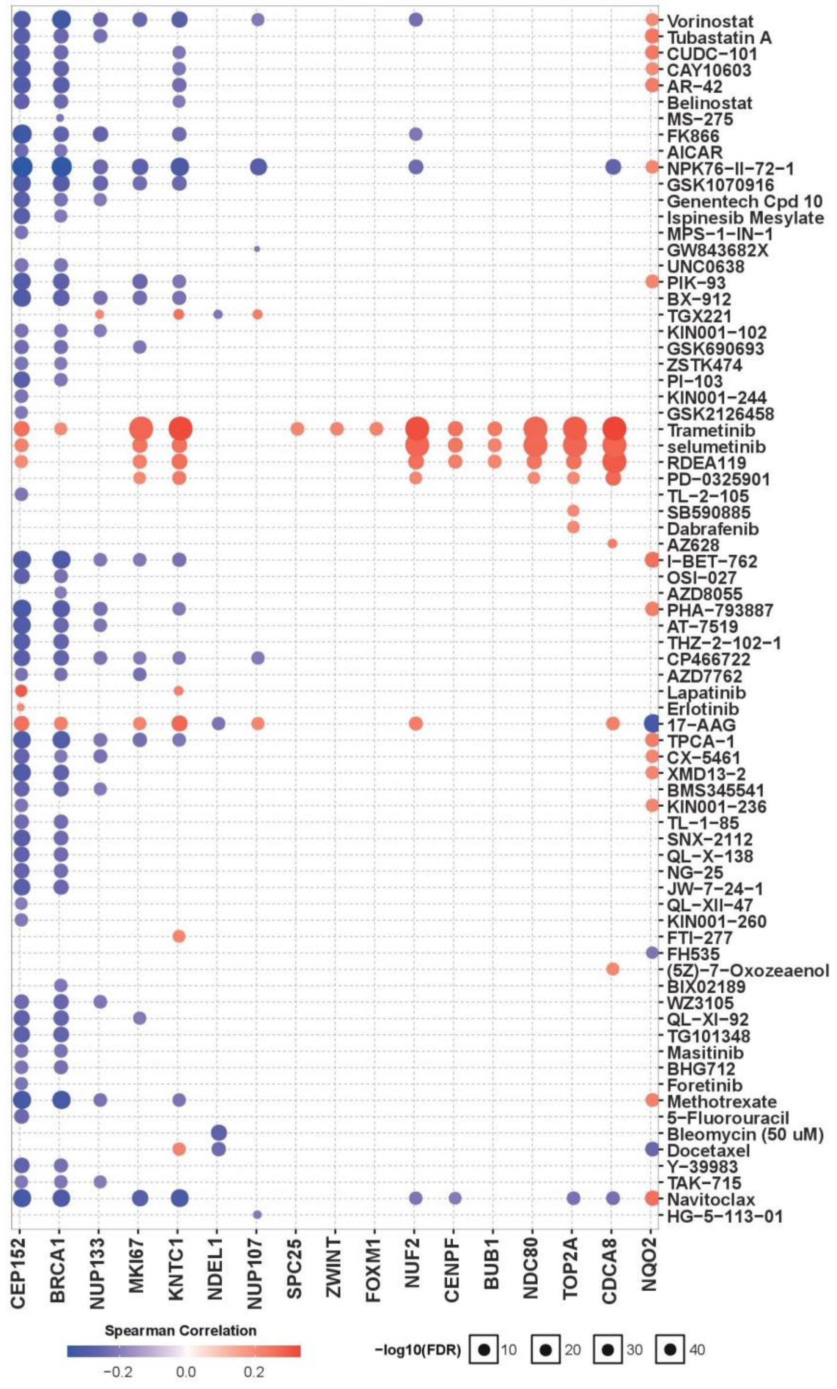

Figure S8. CENPF-related drug sensitivity analysis in sarcoma from GDSC database. Y-axis displayed a list of therapeutic drugs for sarcoma, and X-axis listed CENPF and its co-expressed genes.

Table S1. The relationship between *CENPF* expression and clinicopathological characteristics in LPS patients.

Variables		No. of patient	*CENPF* Expression		X^2	*p* value
			Upper 50%	Lower 50%		
Therapy outcome						
	Progressive Disease	15	8	7	0.04	0.849
	Complete Response	18	9	9		
Recurrence						
	No	14	5	9	2.1	0.147
	Yes	9	6	3		
margin_status						
	Positive	17	7	10	1.06	0.303
	Negative	17	10	7		
Tumor status						
	Tumor free	23	13	10	0.5	0.48
	With tumor	32	15	17		
Tumor size						
	Upper 25%	13	7	6	0.1	0.75
	Lower 75%	41	20	21		
Gender						
	Female	19	9	10	0.08	0.78
	Male	39	20	19		
Age at diagnosis						
	>= 60	39	20	19	0.08	0.78
	< 60	19	9	10		
New tumor after treatment						
	No	31	17	14	0.43	0.514
	Yes	26	12	14		
Tumor multifocal						
	No	33	15	18	0.98	0.322
	Yes	22	13	9		
Metastatic						
	No	20	9	11	1.2	0.273
	Yes	4	3	1		

Table S2. Summary of therapeutic drugs for LPS patients.

Name	Targets	Clinical trial stage	Clinical trial number	Dosage	Mechanism	References (PMID)
Trabectedin	DNA minor groove; FUS-CHOP	Marketed	ET743-SAR3007	1.5 mg/m2	DNA synthesis inhibitors	29220294; 35806460; 27615729
Eribulin	microtubules	Marketed	NCT01327885	1.4 mg/m2	microtubule dynamics inhibitor	29220294; 35546669; 28854066
*Dacarbazine	DNA	Phase III	NCT01327885	850 mg/m2, or 1,000 mg/m2, or 1,200 mg/m2	kylating agents	29220294; 28854066
*Doxorubicin	microtubules	Phase III	NCT02451943	75 mg/m2	topoisomerase II inhibitors, microtubule inhibition	35806460; 32391141; 35547106
Olaratumab	PDGFRα	Registered	NCT02451943	15 mg/ kg or 20 mg/ kg	monoclonal antibody against PDGFRα	32391141; 29413687; 35547106
*Doxorubicin plus Ifosfamide	DNA	Phase III	NCT00061984	D: 75 mg/m2; I: 10 g/m2	DNA-alkylating and cross-linking	18647323; 34881180
*Epirubicin	topoisomerase II	Phase III	NCT01710176	60 mg/m2 daily	topoisomerase II activity inhibition	28493858; 35561319
Milademetan	MDM2	Phase III (ongoing)	NCT04979442	260 mg once daily	oral MDM2 inhibitor	35609512
Abemaciclib	CDK4/6	Phase III (ongoing)	NCT04967521	200 mg twice daily	oral CDK4/6 inhibitor	35609512; 32593094
Selinexor	XPO1, NF-kB	Phase III	NCT02606461	60 mg twice a week	blocks XPO1, inhibits NF-kB pro-oncogenic activity	29220294; 35394800
*Palbociclib	CDK4/6	Phase II	NCT01209598	125 mg daily; 200 mg daily	oral CDK4/6 inhibitor, prevents Rb phosphorylation	29220294; 27124835
Pembrolizumab	PD1	phase II	SARC028	200 mg every three weeks	anti-PD1 antagonists	29220294; 31900276
Efatutazone	PPARγ	Phase II	NCT02249949	0.5 mg twice daily	PPARγ agonist, exerts anticancer activity	29220294; 22570147
*Alisertib	AURKA	Phase II	NCT01653028	50 mg orally b.i.d	oral, ATP-competitive, inhibitor of AURKA	29220294; 27502708
*Docetaxel	microtubules	Phase II	NCT00887809	75 mg/m2	inhibits microtubule network reorganization	35806460; 26074722; 32619151
Pazopanib	tyrosine kinase	Phase II	NCT01506596	800 mg daily	tyrosine kinase inhibitors, angiogenesis inhibitors	29220294; 34050255; 35740650
Ribociclib	CDK4/6	Phase I/II	NCT03096912	600 mg once daily	CDK4/6 inhibitor	29220294; 34921024
*Topotecan	topoisomerase I	Phase II	NCT02357810	8 mg on day1, 8 and 15 (combination with pazopanib)	DNA topoisomerase I inhibitors	34050255; 35725272
Nivolumab	T cell; PD1	Phase II	NCT03307616	3 mg/kg every 2 weeks	PD-1 receptor antagonists; T lymphocyte stimulants	35609512; 30249211
*Ipilimumab	CTLA4	Phase II	NCT03307616	3 mg/kg on week 1	cytotoxic T-lymphocyte antigen 4 inhibitors	30249211

Sunitinib	PDGFR-α and -β; VEGFR1-3; c-Kit; FLT3	Phase I/II	NCT00400569	50 mg daily	multi-targeted tyrosine kinase inhibitor	29220294; 21154746
*Sorafenib	Raf, VEGFR1-3, PDGFRB, FLT3, c-Kit	Phase II	S0505	400 mg twice daily	multi-targeted tyrosine kinase inhibitor	29220294; 21751200
*Regorafenib	KIT, PDGFR, FGFR1, RET, BRAF, VEGFR1–3	Phase II	NCT02048371	160 mg once daily	multi-targeted kinase inhibitor	29220294; 32701199
*Rofecoxib	COX-2	Phase II		25 mg daily	cyclo-oxygenase 2 inhibitors	15470711
Apatinib	VEGFR2, c-Kit, Ret	Phase II	NCT03121846	500mg po qd	VEGFR-2 antagonists	34868934; 31786466
Sitravatinib	c-Met, PDGFR, c-Kit and IGF1R	Phase II	NCT02978859	150 mg/kg daily	receptor tyrosine kinase inhibitors	29220294; 26675259
ATX-101	adipocyte	Phase II	NCT05116683	60 mg/m2 IV weekly	an injectable form of deoxycholic acid, causes adipo-cytolysis when injected subcutaneously into fat	35609512; 27430612
BI 907828	MDM2	Phase II/III	NCT05218499	45 mg	oral MDM2 antagonist	35609512
*Pioglitazone	PPAR-α and -γ	Phase II (ongo-ing)	NCT04794127	45 mg daily	dual agonist of PPAR-α and PPAR-γ	35609512; 15470711
*Plocabulin	tubulin	Phase I		16 mg/kg i.v., QW (mice)	Tubulin polymerisation inhibitors	35806460
*MK-8242	MDM2	Phase I	NCT01463696	400 mg twice a day	MDM2 inhibitors	29220294; 28240971
*AMG 232	MDM2	Phase I	NCT01723020	240 mg	MDM2 inhibitors	35609512; 31359240
Itacitinib	JAK1	Phase I (ongo-ing)	NCT03670069	120 mg/kg orally, b.i.d. (mice)	Janus kinase 1 inhibitor	35609512; 32998963
Seclidemstat	LSD1	Phase I/II (MLPS)	NCT03600649	100 mg/kg/day (mice)	lysine-specific demethylase 1 in-hibitor	35609512; 34453478
SAR405838	MDM2	Phase I (discon-tinued)	NCT01636479	300 mg once daliy	MDM2 inhibitors, activates p53-dependent cell-cycle arrest and apoptosis	29220294; 28324749; 35609512
RG7112	MDM2	Phase I (discon-tinued)	NCT00559533	a starting dose of 20mg/m2/day	MDM2 inhibitors, activates p53 pathway	29220294; 23084521

This table shows the latest development of drugs in references.

*The highest develpoment phase of drugs is not available in the Adisinsight (a drug database).

D: Doxorubicin; I: Ifosfamide
